# Characterising the north-western European species of *Phaenoglyphis* Förster, 1869 (Hymenoptera: Figitidae: Charipinae) with novel insights from DNA barcode data

**DOI:** 10.3897/BDJ.12.e120950

**Published:** 2024-05-20

**Authors:** Jonathan Vogel, Ralph S. Peters, Jesús Selfa, Mar Ferrer-Suay

**Affiliations:** 1 Leibniz Institute for the Analysis of Biodiversity Change, Museum Koenig Bonn, Bonn, Germany Leibniz Institute for the Analysis of Biodiversity Change, Museum Koenig Bonn Bonn Germany; 2 Universitat de València, Valencia, Spain Universitat de València Valencia Spain

**Keywords:** Charipinae, *Phaenoglyphis*, integrative taxonomy, CO1 barcoding, GBOL III: Dark Taxa, new records

## Abstract

**Background:**

The taxonomy of the hymenopteran parasitoid subfamily Charipinae (Hymenoptera: Cynipoidea: Figitidae) has, until recently, been in a state of chaos. While this situation has improved significantly in recent years, most of the efforts were focused on morphological data of typically old specimens. Here, we present the first integrative approach to describe the diversity of the genus *Phaenoglyphis* Förster, 1869 from north-western Europe.

**New information:**

For seven (of a total of 17) species, we provide DNA barcode data. *Phaenoglyphisbelizini* Pujade-Villar, 2018 and *Phaenoglyphisevenhuisi* Pujade-Villar & Paretas-Martínez, 2006 are recorded for the first time from Germany. All DNA barcodes and specimen data were added to the publicly available GBOL and BOLD reference database. The presence of a 6 bp long deletion in the CO1 barcode region that is characteristic to the genus and unique amongst Figitidae supports the monophyly of *Phaenoglyphis*.

## Introduction

Cynipoidea includes phytophagous gall formers or parasitoids of other insects. There are seven recognised families within Cynipoidea: Austrocynipidae, Ibaliidae, Liopteridae, Cynipidae, Diplolepidae, Paraulacidae, and Figitidae (Hearn et al. 2024). Within Figitidae, there are 11 subfamilies, one of them being Charipinae. Charipinae has eight valid genera of which we focus on *Phaenoglyphis* Förster, 1869.

This genus is the second-most diverse within Charipinae. Species are typically smooth, shiny and small with a body length of 0.8 to 2.0 mm, just like their fellow charipines. *Phaenoglyphis*, however, can be easily distinguished by the presence of a mesopleural line and a well-segmented metasoma. Most species possess clearly delineated scutellar foveae and some show slightly to heavily ingrained notauli. The genus currently includes 34 extant species and is found worldwide (Ferrer-Suay et al. 2023). Members of *Phaenoglyphis* are hyperparasitoids of Aphididae (Hemiptera: Aphidoidea) via Aphidiinae (Hymenoptera: Braconidae) and Aphelininae (Hymenoptera: Aphelinidae) (Menke and Evenhuis 1991). By assaulting obligate antagonists of aphids, *Phaenoglyphis*, as well as other charipines, play an important regulatory role in natural systems and in biological control (Müller et al. 1999, Van Veen et al. 2001).

Re-descriptions and a key for all Palaearctic species of *Phaenoglyphis* were published in Ferrer-Suay et al. (2018). In Ferrer-Suay et al. (2021), the status of this genus was revised and more recently, in Ferrer-Suay et al. (2023), data on its diversity and distribution patterns were published. The latest phylogenetic study of Charipinae, which was based on morphological characters alone, inferred *Phaenoglyphis* as a basal, but paraphyletic genus (Paretas-Martínez et al. 2007).

Integrating results from analysis of molecular sequence data is routine in our modern taxonomic toolkit. Sequence data, most notably of the CO1 barcode region, become increasingly attractive in ecological studies and biomonitoring schemes as more and more species are added to publicly available reference libraries (https://data.bolgermany.de/ergebnisse/results (Hausmann et al. 2020) and https://www.boldsystems.org/ (Ratnasingham and Hebert 2007)). This project is part of the German Barcode of Life initiative GBOL III: Dark Taxa which focuses on understudied “dark” insect taxa, including Figitidae (Awad et al. 2020).

Our main objective is to improve the delimitation and characterisation of species within *Phaenoglyphis* in an integrative taxonomy approach. Additionally, we provide new country records as well as a newly-found character pointing towards monophyly of the genus.

## Materials and methods

**Institutional abbreviations**

- EVK – Entomologischer Verein Krefeld e.V., Krefeld, Germany;
- NINA - Norwegian Institute for Nature Research, Trondheim, Norway;
- NTNU - Norwegian University of Science and Technology, University Museum, Department of Natural History, Trondheim, Norway;
- UB - University of Barcelona;
- ZFMK - Leibniz Institute for the Analysis of Biodiversity Change, Museum Koenig Bonn, Germany;
- ZSM - Zoologische Staatssammlung München, Germany.

All specimens with the ‘ZFMK-TIS-...’ tag were prepared at the molecular laboratory of the ZFMK in Bonn in the course of the GBOL III: Dark Taxa project, following the procedures as described in Jafari et al. (2023), using LCO1490-JJ as forward and HCO2198-JJ as reverse primer (Astrin and Stüben 2008). Sequencing was done at BGI (Hong Kong, China). We first assembled forward- and reverse-reads to contig files to then infer consensus sequences using Geneious Prime 2022.1.1 (Biomatters Ltd.). The DNA of specimen ‘HM128-04-CC’ was extracted at the ZFMK using HotSHOT extractions (Truett et al. 2000) by adding 25 µl alkaline lysis buffer and the full body specimen as tissue. We incubated the specimen at 70°C for 30 minutes. We added 25 µl of neutralising solution and used 1 µl of the extract in the PCR. The sample was further processed along with others not used here, using LCO1490-JJ and HCO2198-JJ primers (Astrin and Stüben 2008) with individual tags attached. We sequenced the amplicon pool using MinION technology as described in Vasilita et al. (2024). In addition to the sequences produced in GBOL III: Dark Taxa, we downloaded all *Phaenoglyphis* CO1 sequences of European origin and three *Alloxysta* sequences as outgroup sequences from BOLD (Ratnasingham and Hebert 2007, accessed on 7 Sep 2023). We aligned all sequences using Clustal Omega 1.2.2 (Sievers et al. 2011). All CO1 barcode sequences generated herein and the downloaded sequences with corresponding BOLD-ID are listed in Suppl. material 1.

Using IQ-Tree v.2.2.2.6, we reconstructed a Maximum Likelihood tree by applying the -s option and ultrafast bootstrap with 1000 replicates (Hoang et al. 2018) and without further modifications of parameters (Minh et al. 2020). We performed three molecular species delimitation algorithms on the dataset. Firstly, we applied the ASAP species delimitation algorithm on our alignment (via https://bioinfo.mnhn.fr/abi/public/asap/ accessed 16 Jan 2024, Puillandre et al. (2021)) using default settings. Secondly, we used the Cluster function of Species Identifier v.1.6.2 (Meier et al. 2006) on our alignment, set to a 3% threshold. We chose a 3% threshold as was proposed by Hebert et al. (2003) and supported as objective clustering in Hartop et al. (2022). Lastly, we performed multirate PTP (via https://mptp.h-its.org/#/tree accessed 16 Jan 2024, Kapli et al. (2017)), using default settings on the tree file previously constructed with IQ-Tree. For the tree figure, we combined the final tree and the species delimitation results using InkScape v.1.2 (Inkscape Project).

For the molecular characterisation of species, we used the distance matrix from the alignment provided in Geneious (Suppl. material 2) to extract the maximum intraspecific barcode-distance, stating the sample size in parentheses, and the minimum intraspecific distance, stating the name of the closest species in parentheses. We generated a consensus sequence by aligning the sequences of each species separately in Geneious. As the molecular characterisation is part of an integrative taxonomy approach, we only used the sequences of those specimens that we studied morphologically.

All specimens were left externally intact during DNA extraction and each was point-mounted post lysis using Shellac glue. We attempted to spread the legs and wings so that no other body part was obscured. The metasoma was aligned with the body axis. All specimens are deposited at ZFMK unless stated otherwise.

All specimens were examined using a Leica M205C stereomicroscope (JV & RSP) and Optika ZSM-2 (MFS). Morphological terms and abbreviations are taken from Paretas-Martínez et al. (2007). The width of the fore wing radial cell is measured from the margin of the wing to the beginning Rs vein. Females and males share the same character states, except where indicated.

## Taxon treatments

### Phaenoglyphis

Förster, 1869

E1325346-1CAB-5222-972B-D6318DCE0332

#### Diagnosis

##### Morphological diagnosis

Like in *Alloxysta*, the metasomal tergites are not fused in *Phaenoglyphis*. It is the only genus which has a mesopleural sulcus, which differentiates it from *Alloxysta* and other charipine genera. The presence of notauli and the presence and shape of the scutellar foveae are also unique for many *Phaenoglyphis* species within the charipines (though both notauli and scutellar foveae are absent in some *Phaenoglyphis* species).

##### Molecular diagnosis

**Figure 1.**
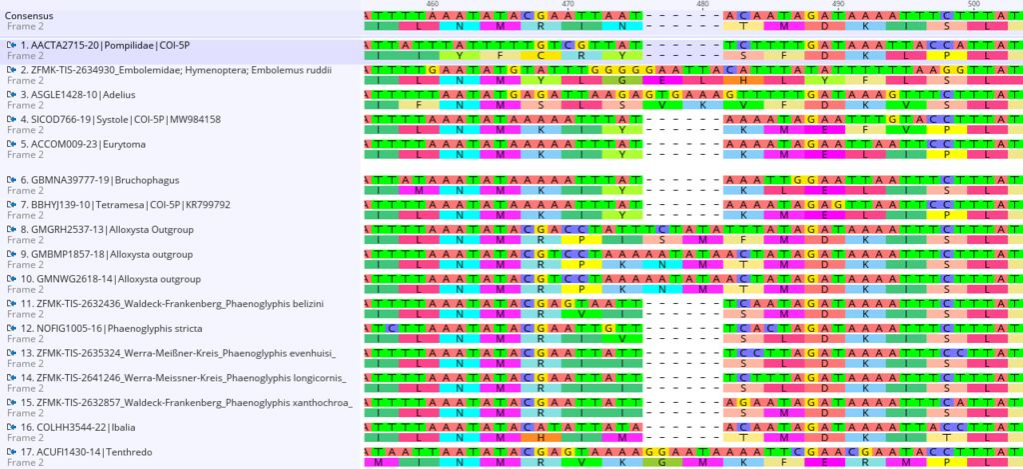
Barcode alignment excerpt showing the 6 bp deletion that is present in all studied *Phaenoglyphis* specimens and species as well as in *Ibalia* (Ibaliidae) and some more distantly-related Hymenoptera taxa. The non-Charipinae sequences were accessed via BOLD and were chosen, based on previously reported gaps in the CO1 barcode region.

A 6 bp deletion is characteristic for the genus *Phaenoglyphis*. It is unique within Figitidae and shared with *Ibalia* Latreille, 1802 (Fig. 1). The two-amino-acid deletion of *Phaenoglyphis* and *Ibalia* is located at the same position as the deletion of a Pompilidae species reported by Park et al. (2010) and of all Eurytomidae reported recently by Jafari et al. (2023). The gap is roughly at bp-position 476 et seqq., though its exact location is difficult to conclude upon as the site is overall very variable. It might be that the gap is, in fact, not homologous with the gap in Pompilidae and Eurytomidae. In other closely-related taxa (*Alloxysta*, *Apocharips* and *Dilyta*), the six here present base pairs code for arguably random amino acids (Fig. 1).

### Phaenoglyphis belizini

Pujade-Villar, 2018

238AFEBD-A0D7-58E8-B615-3B7B5DE2A27E

#### Materials

- **Type status:** Other material. **Occurrence:** recordedBy: GBOL III; individualCount: 1; sex: female; disposition: in collection; occurrenceID: 89B521B3-36C3-527D-BEEC-EE92BCFA83CB; **Taxon:** family: Figitidae; genus: Phaenoglyphis; specificEpithet: belizini; scientificNameAuthorship: Pujade-Villar, 2018; **Location:** country: Germany; countryCode: DE; stateProvince: Hesse; municipality: Waldeck-Frankenberg; locality: National park Kellerwald-Edersee, Maierwiesen; verbatimElevation: 363 m; decimalLatitude: 51.1552; decimalLongitude: 9.0011; **Identification:** identifiedBy: Mar Ferrer-Suay; dateIdentified: 2023; **Event:** eventID: 1034; samplingProtocol: Malaise trap (Krefeld type); eventDate: 2021-6/7-22/8; year: 2018; **Record Level:** language: en; institutionID: ZFMK; collectionID: ZFMK-TIS-2632436; basisOfRecord: PreservedSpecimen
- **Type status:** Other material. **Occurrence:** recordedBy: Kothe, T.;Engelhardt, M.;König, Ch.; individualCount: 1; sex: female; disposition: in collection; occurrenceID: 4C1819E8-BC2D-5A9D-BFB2-85937D258A84; **Taxon:** family: Figitidae; genus: Phaenoglyphis; specificEpithet: *belizini*; scientificNameAuthorship: Pujade-Villar, 2018; **Location:** country: Germany; countryCode: DE; stateProvince: Baden-Württemberg; municipality: Tübingen; locality: Wurmlingen, Gengental; verbatimElevation: 377 m; decimalLatitude: 48.5131; decimalLongitude: 8.9923; **Identification:** identifiedBy: Mar Ferrer-Suay; dateIdentified: 2023; **Event:** eventID: 181; samplingProtocol: Malaise trap; eventDate: 2014-5/6-23/6; year: 2018; **Record Level:** language: en; institutionID: ZFMK; collectionID: ZFMK-TIS-2640663; basisOfRecord: PreservedSpecimen
- **Type status:** Other material. **Occurrence:** recordedBy: GBOL III; individualCount: 1; sex: female; disposition: in collection; occurrenceID: 989AEF1E-93A9-5A88-83F4-309A0EDB5B88; **Taxon:** family: Figitidae; genus: Phaenoglyphis; specificEpithet: *belizini*; scientificNameAuthorship: Pujade-Villar, 2018; **Location:** country: Germany; countryCode: DE; stateProvince: Hesse; municipality: Waldeck-Frankenberg; locality: NP Kellerwald-Edersee, "Maierwiesen"; verbatimElevation: 365 m; decimalLatitude: 51.1555; decimalLongitude: 9.0015; **Identification:** identifiedBy: Mar Ferrer-Suay; dateIdentified: 2023; **Event:** eventID: 1295; samplingProtocol: Malaise trap; eventDate: 2021-6-8/22; year: 2018; **Record Level:** language: en; institutionID: ZFMK; collectionID: ZFMK-TIS-2640974; basisOfRecord: PreservedSpecimen

#### Diagnosis

Female antennae with rhinaria beginning on F3, pedicel longer than F1, F1 slightly longer than F2, F2 shorter than F3 (Fig. 3a); pronotal carinae present, notauli absent, scutellar foveae present, oval, separated by median carina and almost completely defined (upper part with weak impression) (Fig. 4a), propodeal carinae present; radial cell closed, 2.7 times as long as wide.

##### Molecular characterisation

Maximum barcode-distance within species: 2.5% (3).

Minimum barcode-distance to closest species: 6.3% (*P.villosa*)

Consensus barcode sequence (652 bp):

5’-AATTTTATATTTTATTTTTGGAATTTGGTCAGGAATAATTGGATCTGCTTTAAGAATAATTATTCGTATAGAATTAGGAACTCCATCACAACTTATTGGAAATGATCAAATTTATAATTCAATTGTTACAGCTCATGCTTTTATTATAATTTTTTTTATAGTAATACCTATTATAGTTGGAGGATTTGGAAATTATTTAGTTCCTTTAATATTATCAGCTCCAGATATAGCATTTCCTCGTCTTAATAATATAAGATATTGATTATTATTACCAGCTTTAATTTTATTAGTATCAAGAATATTTATTGATCAAGGAGCAGGAACAGGGTGAACAGTCTATCCTCCTTTATCTTCAAATTTAAGACATTCAGGAATTTCTGTTGATTTAACAATTTTTGCTTTACATTTAAGAGGAGTTTCTTCAATTTTAGGAGCTATTAATTTTATTACAACAATTTTAAATATACGAGTAATTTCAATAGATAAAATTTCTTTATTTATTTGATCAATTTTTTTAACAACTATTTTATTATTATTATCTTTACCAGTTTTAGCTGGAGGTATTACAATATTATTATTTGATCGTAATATAAATACTTCATTTTTTGATCCAATAGGAGGAGGGGATCCAATTCTTTACCAACATTTATTT-3’

#### Distribution

China: Beijing province (Ferrer-Suay et al. 2023). **New record** from Germany: Baden-Württemberg, Hesse.

### Phaenoglyphis evenhuisi

Pujade-Villar & Paretas-Martínez, 2006

C4CA10C3-D510-5A5F-8215-DC8EAAA826F8

#### Materials

- **Type status:** Other material. **Occurrence:** recordedBy: Vogel, Jonathan; individualCount: 1; sex: female; disposition: in collection; associatedSequences: GBHYG1732-23; occurrenceID: 4051BAA3-8DFA-513B-81FC-790E2F77BAAD; **Taxon:** family: Figitidae; genus: Phaenoglyphis; specificEpithet: *evenhuisi*; scientificNameAuthorship: Pujade-Villar & Paretas-Martínez, 2006; **Location:** country: Germany; countryCode: DE; stateProvince: Hesse; municipality: Werra-Meißner-Kreis; locality: Großalmerode, Private garden, Siedlerweg; verbatimElevation: 383 m; decimalLatitude: 51.2591; decimalLongitude: 9.7871; **Identification:** identifiedBy: Mar Ferrer-Suay; dateIdentified: 2023; **Event:** eventID: 1632; samplingProtocol: Malaise trap; eventDate: 2022-7-12/20; year: 2006; habitat: semi-abandoned garden with wet spot, ivy hedge and salix; **Record Level:** language: en; institutionID: ZFMK; collectionID: ZFMK-TIS-2635324; basisOfRecord: PreservedSpecimen
- **Type status:** Other material. **Occurrence:** recordedBy: GBOL III; individualCount: 1; sex: female; disposition: in collection; occurrenceID: 21D94939-30F2-57B1-9416-9314701C185E; **Taxon:** family: Figitidae; genus: Phaenoglyphis; specificEpithet: *evenhuisi*; scientificNameAuthorship: Pujade-Villar & Paretas-Martínez, 2006; **Location:** country: Germany; countryCode: DE; stateProvince: Hesse; municipality: Waldeck-Frankenberg; locality: National park Kellerwald-Edersee, Kleiner Mehlberg; verbatimElevation: 370 m; decimalLatitude: 51.2108; decimalLongitude: 9.0428; **Identification:** identifiedBy: Mar Ferrer-Suay; dateIdentified: 2023; **Event:** eventID: 1035; samplingProtocol: Malaise trap (Krefeld type); eventDate: 2021-6/7-22/8; year: 2006; **Record Level:** language: en; institutionID: ZFMK; collectionID: ZFMK-TIS-2632880; basisOfRecord: PreservedSpecimen
- **Type status:** Other material. **Occurrence:** recordedBy: Doczkal, D.; Grabow, K.; individualCount: 1; sex: female; disposition: in collection; occurrenceID: 1A2DA62B-1BC5-5BA0-80C6-A3214EC76E49; **Taxon:** family: Figitidae; genus: Phaenoglyphis; specificEpithet: *evenhuisi*; scientificNameAuthorship: Pujade-Villar & Paretas-Martínez, 2006; **Location:** country: Germany; countryCode: DE; stateProvince: Baden-Württemberg; municipality: Karlsruhe; locality: Malsch, Luderbusch; verbatimElevation: 112 m; decimalLatitude: 48.9144; decimalLongitude: 8.3324; **Identification:** identifiedBy: Mar Ferrer-Suay; dateIdentified: 2023; **Event:** eventID: 864; samplingProtocol: Malaise trap; eventDate: 2020-5-10/17; year: 2006; habitat: salix-populus forest/wild land; **Record Level:** language: en; institutionID: ZFMK; collectionID: ZFMK-TIS-2634989; basisOfRecord: PreservedSpecimen

#### Diagnosis

Female antennae with rhinaria beginning on F4, pedicel shorter than F1, F1 longer than F2, F2 subequal to F3, F3 shorter than F4 (Fig. 3b); notauli present, but weak, scutellar foveae present separated by median carina, each fovea has a transverse posterior carina inside (Fig. 4b); radial cell closed, 3.0 times as long as wide.

##### Molecular characterisation

Maximum barcode-distance within species: 0.3% (3).

Minimum barcode-distance to closest species: 8.9% (*P.longicornis*).

Consensus barcode sequence (652 bp):

5’-TTTATTATATTTTATTTTTGGAATTTGGTCAGGTATAATTGGATCCGCCCTAAGAATAATTATTCGTATAGAATTAGGGACCCCTTCTTCATTAATTGGAAATGATCAAATTTATAATTCAATTGTAACAGCCCACGCTTTTATCATAATTTTTTTTATAGTAATACCTATCATAGTCGGGGGATTTGGTAATTATTTAGTCCCATTAATATTAAGGGCCCCAGATATAGCTTTCCCACGTTTAAATAACATAAGTTTTTGATTATTGCCCCCTGCTTTATTTTTATTAGTTTCTAGAATATTTATTGATCAAGGGGCTGGAACTGGATGAACGGTTTATCCGCCCCTTTCATCTAATTTAGGACATTCAGGAATCTCAGTAGATTTAACTATTTTTTCTTTACATTTAAGAGGTATTTCTTCAATTTTAGGTGCAATTAATTTTATTTCAACAATTTTAAATATACGAATTATTTCCTTAGATAAAATTTCCTTATTTATTTGATCTATTTTTTTAACAACTATTTTATTATTATTATCATTACCTGTATTAGCCGGAGGAATTACAATATTATTATTTGACCGAAATTTAAATACCTCTTTTTTTGACCCTATAGGAGGAGGTGATCCAATTTTATACCAACATTTATTT-3’

#### Distribution

Andorra and France (Ferrer-Suay et al. 2023). **New record** from Germany: Baden-Württemberg, Hesse.

### Phaenoglyphis longicornis

(Hartig, 1840)

214CBB72-97C9-5ECB-8351-33369695CF53

#### Materials

- **Type status:** Other material. **Occurrence:** recordedBy: GBOL III; individualCount: 1; sex: female; disposition: in collection; occurrenceID: D005101E-67AD-5398-BDA3-687A09723391; **Taxon:** family: Figitidae; genus: Phaenoglyphis; specificEpithet: *longicornis*; scientificNameAuthorship: (Hartig, 1840); **Location:** country: Germany; countryCode: DE; stateProvince: Hesse; municipality: Werra-Meißner-Kreis; locality: Frankershausen, Nat. res., "Kripp- und Hielöcher" (Loc. 5.2); verbatimElevation: 302 m; decimalLatitude: 51.2491; decimalLongitude: 9.9198; **Identification:** identifiedBy: Mar Ferrer-Suay; dateIdentified: 2023; **Event:** eventID: 141; samplingProtocol: Malaise trap; eventDate: 2020-10-14/27; year: 1840; habitat: Small Pinus sylvestris forest with lots of dead wood; **Record Level:** language: en; institutionID: ZFMK; collectionID: ZFMK-TIS-2641246; basisOfRecord: PreservedSpecimen

#### Diagnosis

Antennae of both sexes with rhinaria beginning on F1, pedicel shorter than F1, F1 longer than F2, F2 subequal to F3, F3 shorter than F4 (Fig. 3c); pronotal carinae present, notauli present, scutellar foveae oval, with straight anterior and anterolateral margins, separated by median carina, open posteriorly (Fig. 4c), propodeal carinae present; radial cell closed, 2.7 times as long as wide.

##### Molecular characterisation

Maximum barcode-distance within species: not applicable (1).

(Minimum) barcode-distance to closest species: 4.9% (*P.salicis*).

Barcode sequence (652 bp):

5’-TTTATTGTATTTTATTTTTGGAATTTGATCGGGTATAATCGGGTCAGCTTTAAGAATAATTATCCGAATAGAATTAGGAACCCCATCTTCATTAATCGGTAATGATCAAATTTATAATTCAATTGTTACAGCTCATGCTTTTATTATAATTTTTTTTATAGTCATACCAATTATAGTAGGGGGATTTGGAAATTATTTAGTTCCTCTAATATTAAGTGCTCCTGATATAGCTTTCCCACGATTAAATAACATAAGTTTTTGATTATTACCTCCTGCTTTATTTCTATTAATTTCTAGAATATTTATTGATCAAGGGGCTGGAACTGGATGAACTGTTTATCCTCCTCTTTCATCTAATATAGGCCATTCAGGAATTTCAGTAGATTTAACTATTTTTTCTTTACATTTAAGGGGAATTTCTTCTATTTTAGGGGCTATTAATTTTATTTCAACAATTTTAAATATACGAATTATTTCTTTAGATAAAATTTCTTTATTTATTTGATCTATTTTTTTAACAACTATTTTATTATTATTATCATTACCTGTATTAGCAGGAGGAATTACTATATTATTATTTGATCGAAATTTAAATACTTCTTTTTTTGATCCAATGGGAGGGGGAGACCCTATTTTATATCAACATTTATTT-3’

#### Distribution

France, Germany, India, Romania, Spain, Sweden and United Kingdom: England, Scotland (Ferrer-Suay et al. 2023).

#### Taxon discussion

The species *P.salicis* and *P.longicornis* were inferred as conspecific only in the second-ranked partition in analyses with ASAP, but separate in all others, which is in line with our morphological concept of the two species.

### Phaenoglyphis salicis

(Cameron, 1883)

BEAC6B79-6294-5609-B443-84044EDD25AB

#### Materials

- **Type status:** Other material. **Occurrence:** recordedBy: Vogel, Jonathan; individualCount: 1; sex: female; disposition: in collection; occurrenceID: CAE83B1F-A869-546B-986C-B850D5B50415; **Taxon:** family: Figitidae; genus: Phaenoglyphis; specificEpithet: *salicis*; scientificNameAuthorship: (Cameron, 1883); **Location:** country: Germany; countryCode: DE; stateProvince: North Rhine-Westphalia; municipality: Rhein-Sieg-Kreis; locality: Alfter, playground next to "KiTa Rasselbande"; verbatimElevation: 86 m; decimalLatitude: 50.7343; decimalLongitude: 7.0138; **Identification:** identifiedBy: Mar Ferrer-Suay; dateIdentified: 2023; **Event:** eventID: 1514; samplingProtocol: hand picked; eventDate: 2022-5-5; year: 1883; habitat: among wood chips on ground; **Record Level:** language: en; institutionID: ZFMK; collectionID: ZFMK-TIS-2634968; basisOfRecord: PreservedSpecimen
- **Type status:** Other material. **Occurrence:** recordedBy: DINA; individualCount: 1; sex: female; disposition: in collection; occurrenceID: 87B29A24-80BF-5955-AF20-8ED77C8898E3; **Taxon:** family: Figitidae; genus: Phaenoglyphis; specificEpithet: *salicis*; scientificNameAuthorship: (Cameron, 1883); **Location:** country: Germany; countryCode: DE; stateProvince: Rhineland-Palatinate; municipality: Cochem; locality: Nat. res. Brauselay; verbatimElevation: 94 m; decimalLatitude: 50.1416; decimalLongitude: 7.1875; **Identification:** identifiedBy: Mar Ferrer-Suay; dateIdentified: 2023; **Event:** eventID: 919; samplingProtocol: Malaise trap; eventDate: 2020-5-29; year: 1883; **Record Level:** language: en; institutionID: EVK; collectionID: ZFMK-TIS-2641264; basisOfRecord: PreservedSpecimen

#### Diagnosis

Antennae of both sexes with rhinaria beginning on F3, pedicel shorter than F1, F1 longer than F2, F2 shorter than F3, F3 subequal to F4 (Fig. 3d); pronotal carinae present, notauli weak, scutellar foveae oval, completely defined and with two lines anteriorly (Fig. 4d), propodeal carinae present; radial cell closed, 2.5 times as long as wide.

##### Molecular characterisation

Maximum barcode-distance within species: 0.2% (2).

(Minimum) barcode-distance to closest species: 4.9% (*P.longicornis)*.

Consensus barcode sequence (652 bp):

5’-TTTATTGTATTTTATTTTTGGAATTTGATCAGGAATAATTGGATCAGCTTTAAGAATAATTATTCGAATAGAATTAGGCACCCCATCTTCATTAATTGGTAATGACCAAATTTATAATTCAATTGTTACAGCTCATGCTTTTATTATAATTTTTTTTATAGTTATACCAATTATAGTAGGAGGATTCGGTAATTATTTAGTTCCTTTAATATTAAGGGCTCCTGATATAGCTTTCCCACGATTAAACAATATAAGTTTTTGATTATTACCCCCCGCTTTATTTTTATTAACTTCTAGAATATTTATTGATCAAGGAGCTGGAACTGGATGAACTGTTTAYCCACCTCTCTCCTCTAATTTAGGCCATTCAGGGATTTCAGTAGATTTAACTATTTTTTCTTTACATTTAAGGGGAATTTCTTCTATTTTAGGAGCTATTAATTTTATTTCAACAATTTTAAATATACGAATTATTTCTTTAGATAAAATTTCTTTATTTATCTGATCTATTTTTTTAACAACTATTTTATTATTATTATCATTACCTGTATTAGCAGGAGGGATCACTATATTATTATTTGATCGAAATTTAAATACTTCTTTTTTTGATCCAATGGGAGGAGGAGACCCTATTTTATACCAACATTTATTT-3’

#### Distribution

Austria, Germany, Ireland, Italy, Romania, Spain, USA: Colorado, and United Kingdom: England, Scotland, Wales (Ferrer-Suay et al. 2014, Ferrer-Suay et al. 2023).

#### Taxon discussion

See the taxon discussion of *P.longicornis*.

### Phaenoglyphis stricta

(Thomson, 1877)

29319A64-4A17-589C-A4C6-2ED13F0769EE

#### Materials

- **Type status:** Other material. **Occurrence:** recordedBy: Sv.Thygeson & Endrestol; individualCount: 1; sex: male; disposition: in collection; associatedSequences: NOFIG1005-16; occurrenceID: EF4EB66E-EA90-5319-A693-2C385B87F602; **Taxon:** family: Figitidae; genus: Phaenoglyphis; specificEpithet: *stricta*; scientificNameAuthorship: (Thomson, 1877); **Location:** country: Norway; countryCode: NO; stateProvince: Aust-Agder; municipality: Froland; locality: Jurdalsknuten; verbatimElevation: 330 m; decimalLatitude: 58.6210; decimalLongitude: 8.2450; **Identification:** identifiedBy: Mar Ferrer-Suay; dateIdentified: 2023; **Event:** eventDate: 2010-8-19; year: 1877; **Record Level:** language: en; institutionID: NINA; collectionID: NOFIG883; basisOfRecord: PreservedSpecimen
- **Type status:** Other material. **Occurrence:** recordedBy: Odegaard, Frode; individualCount: 1; sex: female; disposition: in collection; associatedSequences: NOFIG732-16; occurrenceID: 63646654-B360-5216-8441-B78FD3D7B2F9; **Taxon:** family: Figitidae; genus: Phaenoglyphis; specificEpithet: *stricta*; scientificNameAuthorship: (Thomson, 1877); **Location:** country: Norway; countryCode: NO; stateProvince: Ostfold; municipality: Hvaler; locality: Soendre Sandoey; verbatimElevation: 14 m; decimalLatitude: 59.0080; decimalLongitude: 11.0830; **Identification:** identifiedBy: Mar Ferrer-Suay; dateIdentified: 2023; **Event:** eventDate: 2014-4-25; year: 1877; **Record Level:** language: en; institutionID: NINA; collectionID: NOFIG610; basisOfRecord: PreservedSpecimen

#### Diagnosis

Antennae of both sexes with rhinaria beginning on the last two thirds of F1, pedicel shorter than F1, F1 longer than F2, F2-F4 subequal in length (Fig. 3e); notauli present, scutellar foveae straight laterally, open anteriorly and posteriorly (Fig. 4e).

##### Molecular characterisation

Maximum barcode-distance within species: 0.9% (2).

Minimum barcode-distance to closest species: 12% (*P.xanthochroa*).

Consensus barcode sequence (652 bp):

5’-GATATTATATTTTATTTTTGGTGTGTGATCTGGAATAATTGGGTCATCTTTAAGATTAATTATTCGAATAGAATTAGGAACACCAAACCAATTAATCGGAAATGATCAAATTTATAATTCTATTGTTACTGCYCATGCTTTTATTATAATTTTTTTTATAGTTATACCTATTATAGTAGGAGGGTTTGGTAATTATTTAATTCCTTTAATATTATCCGCCCCCGATATAGCTTTCCCTCGTTTAAATAATATAAGATTTTGACTTTTACCTCCTGCTTTATTATTATTAACATCTAGAATATTTATTGATCAAGGGGCTGGAACAGGGTGAACAGTGTATCCTCCTTTATCATCTAATTTAGGTCATTCAGGYATTGCAGTTGATTTAACAATTTTTTCTTTACATATAAGAGGAATTTCATCAATTTTAGGGTCAATTAATTTTATTACAACAATCTTAAATATACGAATTGTTTCAYTAGATAAAATTTCTTTATTTATTTGATCCATTTTTTTAACAACAATTTTATTGTTATTATCTTTACCAGTATTAGCTGGAGGTATTACTATATTACTTTTTGATCGAAATTTAAATACYTCTTTTTTTGACCCTATAGGAGGAGGRGATCCTATTTTATAYCAACATTTATTT-3’

#### Distribution

Andorra, Denmark, Finland, France, Germany, Mexico: Mexico City, Norway, Russia: Murmansk Oblast, Sweden, United Kingdom: England, and USA: Arizona, Iowa (Hellén 1963, Ferrer-Suay et al. 2013, Ferrer-Suay et al. 2014, Ferrer-Suay et al. 2023).

#### Taxon discussion

The specimens with their corresponding barcodes and identification were published prior to this study on BOLD. Though its occurrence in Norway has not been published in a scientific journal, we refrain from claiming to be the first to record the species for Norway, as this information was publicly available prior to this study.

### Phaenoglyphis villosa

(Hartig, 1841)

A65A318A-B952-5C63-8AD6-5C0787B3CAA7

#### Materials

- **Type status:** Other material. **Occurrence:** recordedBy: Salden, Tobias; individualCount: 1; sex: male; disposition: in collection; associatedSequences: GBHYG1718-23; occurrenceID: 15BA6329-6566-54F2-AE00-DB2A35B95466; **Taxon:** family: Figitidae; genus: Phaenoglyphis; specificEpithet: *villosa*; scientificNameAuthorship: (Hartig, 1841); **Location:** country: Germany; countryCode: DE; stateProvince: North Rhine-Westphalia; municipality: Bonn; locality: ZFMK; verbatimElevation: 67 m; decimalLatitude: 50.7214; decimalLongitude: 7.1139; **Identification:** identifiedBy: Mar Ferrer-Suay; dateIdentified: 2023; **Event:** eventID: 1766; samplingProtocol: sweep net; eventDate: 2022-10-4; year: 1841; habitat: garden; **Record Level:** language: en; institutionID: ZFMK; collectionID: ZFMK-TIS-2635310; basisOfRecord: PreservedSpecimen
- **Type status:** Other material. **Occurrence:** recordedBy: Salden, Tobias; individualCount: 1; sex: male; disposition: in collection; associatedSequences: GBHYG1719-23; occurrenceID: 53FE4D5B-EE46-5EA1-B0EA-E1E86F899E5E; **Taxon:** family: Figitidae; genus: Phaenoglyphis; specificEpithet: *villosa*; scientificNameAuthorship: (Hartig, 1841); **Location:** country: Germany; countryCode: DE; stateProvince: North Rhine-Westphalia; municipality: Bonn; locality: ZFMK; verbatimElevation: 67 m; decimalLatitude: 50.7214; decimalLongitude: 7.1139; **Identification:** identifiedBy: Mar Ferrer-Suay; dateIdentified: 2023; **Event:** eventID: 1766; samplingProtocol: sweep net; eventDate: 2022-10-4; year: 1841; habitat: garden; **Record Level:** language: en; institutionID: ZFMK; collectionID: ZFMK-TIS-2635311; basisOfRecord: PreservedSpecimen
- **Type status:** Other material. **Occurrence:** recordedBy: ZFMK et al.; individualCount: 1; sex: female; disposition: in collection; associatedSequences: GBHYG1782-23; occurrenceID: 46C44CF6-D965-5B64-B9BF-AFC1FEF77459; **Taxon:** family: Figitidae; genus: Phaenoglyphis; specificEpithet: *villosa*; scientificNameAuthorship: (Hartig, 1841); **Location:** country: Germany; countryCode: DE; stateProvince: North Rhine-Westphalia; municipality: Rhein-Sieg-Kreis; locality: Schladern near Windeck, Sieg river, right river bank; verbatimElevation: 131 m; decimalLatitude: 50.8000; decimalLongitude: 7.5850; **Identification:** identifiedBy: Mar Ferrer-Suay; dateIdentified: 2023; **Event:** eventID: 35; samplingProtocol: Malaise trap; eventDate: 2017-6-13/20; year: 1841; **Record Level:** language: en; institutionID: ZFMK; collectionID: ZFMK-TIS-2628162; basisOfRecord: PreservedSpecimen
- **Type status:** Other material. **Occurrence:** recordedBy: Remschak; individualCount: 1; sex: female; disposition: in collection; associatedSequences: GBHYG1874-23; occurrenceID: BB7FED16-1BF5-5B5F-BF31-DF061C1BABD2; **Taxon:** family: Figitidae; genus: Phaenoglyphis; specificEpithet: *villosa*; scientificNameAuthorship: (Hartig, 1841); **Location:** country: Austria; countryCode: AT; municipality: NP Gesäuse; locality: Gsengquelle; verbatimElevation: 683 m; decimalLatitude: 47.5683; decimalLongitude: 14.5902; **Identification:** identifiedBy: Mar Ferrer-Suay; dateIdentified: 2023; **Event:** eventID: 1424; samplingProtocol: sweep net; eventDate: 2015-7-10; year: 1841; **Record Level:** language: en; institutionID: ZFMK; collectionID: ZFMK-TIS-2635167; basisOfRecord: PreservedSpecimen
- **Type status:** Other material. **Occurrence:** recordedBy: Schwingeler, Josefine;Vogel, Jonathan; individualCount: 1; sex: male; disposition: in collection; associatedSequences: LIBNP001-23; occurrenceID: AC483528-0A8B-57B0-B450-B4CBB823B8E7; **Taxon:** family: Figitidae; genus: Phaenoglyphis; specificEpithet: *villosa*; scientificNameAuthorship: (Hartig, 1841); **Location:** country: Germany; countryCode: DE; stateProvince: North Rhine-Westphalia; municipality: Bonn; locality: Garden of Museum Koenig; verbatimElevation: 67 m; decimalLatitude: 50.7215; decimalLongitude: 7.1137; **Identification:** identifiedBy: Mar Ferrer-Suay; dateIdentified: 2023; **Event:** eventID: 1392; samplingProtocol: sweep net; eventDate: 2022-7-4; year: 1841; habitat: Various habitats; **Record Level:** language: en; institutionID: ZFMK; collectionID: HM128-04-CC; basisOfRecord: PreservedSpecimen
- **Type status:** Other material. **Occurrence:** recordedBy: Vogel, Jonathan; individualCount: 1; sex: female; disposition: in collection; occurrenceID: 2C6471CC-548E-5C9C-8BDF-32267C8050C2; **Taxon:** family: Figitidae; genus: Phaenoglyphis; specificEpithet: *villosa*; scientificNameAuthorship: (Hartig, 1841); **Location:** country: Sweden; countryCode: SE; stateProvince: Uppland; municipality: Stockholm; locality: Stora skuggan; verbatimElevation: 21 m; decimalLatitude: 59.3650; decimalLongitude: 18.0800; **Identification:** identifiedBy: Mar Ferrer-Suay; dateIdentified: 2023; **Event:** eventID: 910; samplingProtocol: sweep net; eventDate: 2021-8-31; year: 1841; **Record Level:** language: en; institutionID: ZFMK; collectionID: ZFMK-TIS-2632498; basisOfRecord: PreservedSpecimen
- **Type status:** Other material. **Occurrence:** recordedBy: ZFMK et al.; individualCount: 1; sex: female; disposition: in collection; occurrenceID: 171B7091-428B-5444-8072-4FCF9770275F; **Taxon:** family: Figitidae; genus: Phaenoglyphis; specificEpithet: *villosa*; scientificNameAuthorship: (Hartig, 1841); **Location:** country: Germany; countryCode: DE; stateProvince: North Rhine-Westphalia; municipality: Rhein-Sieg-Kreis; locality: Schladern near Windeck, Sieg river, right river bank; verbatimElevation: 131 m; decimalLatitude: 50.8000; decimalLongitude: 7.5850; **Identification:** identifiedBy: Mar Ferrer-Suay; dateIdentified: 2023; **Event:** eventID: 37; samplingProtocol: Malaise trap; eventDate: 2017-5-23/30; year: 1841; **Record Level:** language: en; institutionID: ZFMK; collectionID: ZFMK-TIS-2632692; basisOfRecord: PreservedSpecimen
- **Type status:** Other material. **Occurrence:** recordedBy: Van Steenis, Jeroen; individualCount: 1; sex: female; disposition: in collection; occurrenceID: 57243196-E6EE-552E-B41D-0F2C8C106AF0; **Taxon:** family: Figitidae; genus: Phaenoglyphis; specificEpithet: *villosa*; scientificNameAuthorship: (Hartig, 1841); **Location:** country: Iceland; countryCode: IS; stateProvince: 1; municipality: Gardðabær; locality: Vífilsstaðavatn; verbatimElevation: 40 m; decimalLatitude: 64.0700; decimalLongitude: -21.8800; **Identification:** identifiedBy: Mar Ferrer-Suay; dateIdentified: 2023; **Event:** eventID: 1230; samplingProtocol: Malaise trap; eventDate: 2021-7-13/29; year: 1841; habitat: lake shore; **Record Level:** language: en; institutionID: ZFMK; collectionID: ZFMK-TIS-2634650; basisOfRecord: PreservedSpecimen
- **Type status:** Other material. **Occurrence:** recordedBy: Vogel, Jonathan; individualCount: 1; sex: male; disposition: in collection; occurrenceID: C02697B2-F437-5666-83DE-2FBC8C1ED7E2; **Taxon:** family: Figitidae; genus: Phaenoglyphis; specificEpithet: *villosa*; scientificNameAuthorship: (Hartig, 1841); **Location:** country: Germany; countryCode: DE; stateProvince: Saxony; municipality: Leipzig; locality: Lake Störmthal (southern bank); verbatimElevation: 117 m; decimalLatitude: 51.2278; decimalLongitude: 12.4566; **Identification:** identifiedBy: Mar Ferrer-Suay; dateIdentified: 2023; **Event:** eventID: 1007; samplingProtocol: sweep net; eventDate: 2021-7-13; year: 1841; **Record Level:** language: en; institutionID: ZFMK; collectionID: ZFMK-TIS-2634971; basisOfRecord: PreservedSpecimen
- **Type status:** Other material. **Occurrence:** recordedBy: DINA; individualCount: 1; sex: female; disposition: in collection; occurrenceID: 50EF98FF-58C2-5C81-9B41-C09575ECE50E; **Taxon:** family: Figitidae; genus: Phaenoglyphis; specificEpithet: *villosa*; scientificNameAuthorship: (Hartig, 1841); **Location:** country: Germany; countryCode: DE; stateProvince: Saxony-Anhalt; municipality: Saalekreis; locality: Nat. res. "Porphyrlandschaft bei Gimritz"; verbatimElevation: 114 m; decimalLatitude: 51.5593; decimalLongitude: 11.8446; **Identification:** identifiedBy: Mar Ferrer-Suay; dateIdentified: 2023; **Event:** eventID: 1073; samplingProtocol: Malaise trap; eventDate: 2020-6-2; year: 1841; **Record Level:** language: en; institutionID: EVK; collectionID: ZFMK-TIS-2635208; basisOfRecord: PreservedSpecimen
- **Type status:** Other material. **Occurrence:** recordedBy: Doczkal, D.;Voith, J.; individualCount: 1; sex: female; disposition: in collection; occurrenceID: 679C05FC-6D7A-5EE8-83FA-5F2D01F0C4D1; **Taxon:** family: Figitidae; genus: Phaenoglyphis; specificEpithet: *villosa*; scientificNameAuthorship: (Hartig, 1841); **Location:** country: Germany; countryCode: DE; stateProvince: Bavaria; municipality: Garmisch-Partenkirchen; locality: Zugspitze; verbatimElevation: 1965 m; decimalLatitude: 47.4062; decimalLongitude: 11.0095; **Identification:** identifiedBy: Mar Ferrer-Suay; dateIdentified: 2023; **Event:** eventID: 2334; samplingProtocol: Malaise trap; eventDate: 2018-7/8-18/2; year: 1841; habitat: mountain; **Record Level:** language: en; institutionID: ZFMK; collectionID: ZFMK-TIS-2637886; basisOfRecord: PreservedSpecimen
- **Type status:** Other material. **Occurrence:** recordedBy: Doczkal, D.;Voith, J.; individualCount: 1; sex: female; disposition: in collection; occurrenceID: 7E3396F2-CC52-5B99-B010-96CA618D498F; **Taxon:** family: Figitidae; genus: Phaenoglyphis; specificEpithet: *villosa*; scientificNameAuthorship: (Hartig, 1841); **Location:** country: Germany; countryCode: DE; stateProvince: Bavaria; municipality: Garmisch-Partenkirchen; locality: Zugspitze; verbatimElevation: 2005 m; decimalLatitude: 47.4068; decimalLongitude: 11.0080; **Identification:** identifiedBy: Mar Ferrer-Suay; dateIdentified: 2023; **Event:** eventID: 2339; samplingProtocol: Malaise trap; eventDate: 2018-8/9-13/11; year: 1841; habitat: mountain; **Record Level:** language: en; institutionID: ZFMK; collectionID: ZFMK-TIS-2637887; basisOfRecord: PreservedSpecimen
- **Type status:** Other material. **Occurrence:** recordedBy: Doczkal, D.;Voith, J.; individualCount: 1; sex: female; disposition: in collection; occurrenceID: 662BA65B-2C72-508D-AF8F-E92457B30BF6; **Taxon:** family: Figitidae; genus: Phaenoglyphis; specificEpithet: *villosa*; scientificNameAuthorship: (Hartig, 1841); **Location:** country: Germany; countryCode: DE; stateProvince: Bavaria; municipality: Garmisch-Partenkirchen; locality: Zugspitze; verbatimElevation: 1965 m; decimalLatitude: 47.4062; decimalLongitude: 11.0095; **Identification:** identifiedBy: Mar Ferrer-Suay; dateIdentified: 2023; **Event:** eventID: 2334; samplingProtocol: Malaise trap; eventDate: 2018-7/8-18/2; year: 1841; habitat: mountain; **Record Level:** language: en; institutionID: ZFMK; collectionID: ZFMK-TIS-2637889; basisOfRecord: PreservedSpecimen
- **Type status:** Other material. **Occurrence:** recordedBy: Doczkal, D.;Voith, J.; individualCount: 1; sex: male; disposition: in collection; occurrenceID: E66C422C-AD47-5816-BB3B-AD313CCA5602; **Taxon:** family: Figitidae; genus: Phaenoglyphis; specificEpithet: *villosa*; scientificNameAuthorship: (Hartig, 1841); **Location:** country: Germany; countryCode: DE; stateProvince: Bavaria; municipality: Garmisch-Partenkirchen; locality: Zugspitze; verbatimElevation: 1965 m; decimalLatitude: 47.4062; decimalLongitude: 11.0095; **Identification:** identifiedBy: Mar Ferrer-Suay; dateIdentified: 2023; **Event:** eventID: 2341; samplingProtocol: Malaise trap; eventDate: 2018-9/10-11/9; year: 1841; habitat: mountain; **Record Level:** language: en; institutionID: ZFMK; collectionID: ZFMK-TIS-2638011; basisOfRecord: PreservedSpecimen
- **Type status:** Other material. **Occurrence:** recordedBy: Doczkal, D.;Voith, J.; individualCount: 1; sex: female; disposition: in collection; occurrenceID: 754FA597-6BF1-55D2-9769-5AB764824C95; **Taxon:** family: Figitidae; genus: Phaenoglyphis; specificEpithet: *villosa*; scientificNameAuthorship: (Hartig, 1841); **Location:** country: Germany; countryCode: DE; stateProvince: Bavaria; municipality: Garmisch-Partenkirchen; locality: Zugspitze; verbatimElevation: 2005 m; decimalLatitude: 47.4068; decimalLongitude: 11.0080; **Identification:** identifiedBy: Mar Ferrer-Suay; dateIdentified: 2023; **Event:** eventID: 2339; samplingProtocol: Malaise trap; eventDate: 2018-8/9-13/11; year: 1841; habitat: mountain; **Record Level:** language: en; institutionID: ZFMK; collectionID: ZFMK-TIS-2638012; basisOfRecord: PreservedSpecimen
- **Type status:** Other material. **Occurrence:** recordedBy: Doczkal, D.;Voith, J.; individualCount: 1; sex: female; disposition: in collection; occurrenceID: D0A827CC-2964-5C57-B2F2-3D941E35329D; **Taxon:** family: Figitidae; genus: Phaenoglyphis; specificEpithet: *villosa*; scientificNameAuthorship: (Hartig, 1841); **Location:** country: Germany; countryCode: DE; stateProvince: Bavaria; municipality: Schwandorf; locality: Bodenwöhr, Postlohe; verbatimElevation: 360 m; decimalLatitude: 49.2760; decimalLongitude: 12.3507; **Identification:** identifiedBy: Mar Ferrer-Suay; dateIdentified: 2023; **Event:** eventID: 881; samplingProtocol: Malaise trap; eventDate: 2016-6-6/25; year: 1841; habitat: forest hamletmarshland forest; **Record Level:** language: en; institutionID: ZFMK; collectionID: ZFMK-TIS-2640634; basisOfRecord: PreservedSpecimen
- **Type status:** Other material. **Occurrence:** recordedBy: Schwingeler, Josefine;Vogel, Jonathan; individualCount: 1; sex: male; disposition: in collection; occurrenceID: 67E5DC82-3C54-5909-88BD-98CB237AF51C; **Taxon:** family: Figitidae; genus: Phaenoglyphis; specificEpithet: *villosa*; scientificNameAuthorship: (Hartig, 1841); **Location:** country: Germany; countryCode: DE; stateProvince: North Rhine-Westphalia; municipality: Bonn; locality: Garden of Museum Koenig; verbatimElevation: 67 m; decimalLatitude: 50.7215; decimalLongitude: 7.1137; **Identification:** identifiedBy: Mar Ferrer-Suay; dateIdentified: 2023; **Event:** eventID: 1392; samplingProtocol: sweep net; eventDate: 2022-7-4; year: 1840; habitat: Various habitats; **Record Level:** language: en; institutionID: ZFMK; collectionID: ZFMK-TIS-2640976; basisOfRecord: PreservedSpecimen
- **Type status:** Other material. **Occurrence:** recordedBy: Schwingeler, Josefine;Vogel, Jonathan; individualCount: 1; sex: male; disposition: in collection; occurrenceID: 29634819-6E30-5986-BB5B-3B002865BE05; **Taxon:** family: Figitidae; genus: Phaenoglyphis; specificEpithet: *villosa*; scientificNameAuthorship: (Hartig, 1841); **Location:** country: Germany; countryCode: DE; stateProvince: North Rhine-Westphalia; municipality: Bonn; locality: Garden of Museum Koenig; verbatimElevation: 67 m; decimalLatitude: 50.7215; decimalLongitude: 7.1137; **Identification:** identifiedBy: Mar Ferrer-Suay; dateIdentified: 2023; **Event:** eventID: 1392; samplingProtocol: sweep net; eventDate: 2022-7-4; year: 1841; habitat: Various habitats; **Record Level:** language: en; institutionID: ZFMK; collectionID: ZFMK-TIS-2640977; basisOfRecord: PreservedSpecimen
- **Type status:** Other material. **Occurrence:** recordedBy: Schwingeler, Josefine;Vogel, Jonathan; individualCount: 1; sex: male; disposition: in collection; occurrenceID: 928C059C-F999-5646-AE03-2DD5FBF81B51; **Taxon:** family: Figitidae; genus: Phaenoglyphis; specificEpithet: *villosa*; scientificNameAuthorship: (Hartig, 1841); **Location:** country: Germany; countryCode: DE; stateProvince: North Rhine-Westphalia; municipality: Bonn; locality: Garden of Museum Koenig; verbatimElevation: 67 m; decimalLatitude: 50.7215; decimalLongitude: 7.1137; **Identification:** identifiedBy: Mar Ferrer-Suay; dateIdentified: 2023; **Event:** eventID: 1392; samplingProtocol: sweep net; eventDate: 2022-7-4; year: 1841; habitat: Various habitats; **Record Level:** language: en; institutionID: ZFMK; collectionID: ZFMK-TIS-2640978; basisOfRecord: PreservedSpecimen
- **Type status:** Other material. **Occurrence:** recordedBy: Schwingeler, Josefine;Vogel, Jonathan; individualCount: 1; sex: female; disposition: in collection; occurrenceID: 803A7FF9-63BB-56A6-839B-236D6F9E14E5; **Taxon:** family: Figitidae; genus: Phaenoglyphis; specificEpithet: *villosa*; scientificNameAuthorship: (Hartig, 1841); **Location:** country: Germany; countryCode: DE; stateProvince: North Rhine-Westphalia; municipality: Bonn; locality: Garden of Museum Koenig; verbatimElevation: 67 m; decimalLatitude: 50.7215; decimalLongitude: 7.1137; **Identification:** identifiedBy: Mar Ferrer-Suay; dateIdentified: 2023; **Event:** eventID: 1392; samplingProtocol: sweep net; eventDate: 2022-7-4; year: 1841; habitat: Various habitats; **Record Level:** language: en; institutionID: ZFMK; collectionID: ZFMK-TIS-2640979; basisOfRecord: PreservedSpecimen
- **Type status:** Other material. **Occurrence:** recordedBy: Krogmann, Lars; individualCount: 1; sex: female; disposition: in collection; occurrenceID: AAE1D554-C1E5-55D0-853D-9DC9580E63D6; **Taxon:** family: Figitidae; genus: Phaenoglyphis; specificEpithet: *villosa*; scientificNameAuthorship: (Hartig, 1841); **Location:** country: Germany; countryCode: DE; stateProvince: Lower Saxony; municipality: Lüchow-Dannenberg; locality: Pevestorf, Deichvorland & Deich; verbatimElevation: 22 m; decimalLatitude: 53.0636; decimalLongitude: 11.4742; **Identification:** identifiedBy: Mar Ferrer-Suay; dateIdentified: 2023; **Event:** eventID: 159; samplingProtocol: Malaise trap; eventDate: 2013-8-6/10; year: 1841; **Record Level:** language: en; institutionID: ZFMK; collectionID: ZFMK-TIS-2641188; basisOfRecord: PreservedSpecimen
- **Type status:** Other material. **Occurrence:** recordedBy: Gilgenbach, Carolin; individualCount: 1; sex: male; disposition: in collection; occurrenceID: 5C1E7871-320D-59A0-A5F2-B90F4680DA4B; **Taxon:** family: Figitidae; genus: Phaenoglyphis; specificEpithet: *villosa*; scientificNameAuthorship: (Hartig, 1841); **Location:** country: Germany; countryCode: DE; stateProvince: Rhineland-Palatinate; municipality: Alzey-Worms; locality: Wine fields north of Monsheim; verbatimElevation: 145 m; decimalLatitude: 49.6406; decimalLongitude: 8.2137; **Identification:** identifiedBy: Mar Ferrer-Suay; dateIdentified: 2023; **Event:** eventID: 1101; samplingProtocol: Malaise trap; eventDate: 2021-8-5/24; year: 1841; habitat: shrub islands between wine fields, mostly poplars; **Record Level:** language: en; institutionID: ZFMK; collectionID: ZFMK-TIS-2641198; basisOfRecord: PreservedSpecimen
- **Type status:** Other material. **Occurrence:** recordedBy: Doczkal, Dieter;Grabow, K.; individualCount: 1; sex: female; disposition: in collection; occurrenceID: EF5B377A-B79B-5D1D-8995-6323F8F6CFC2; **Taxon:** family: Figitidae; genus: Phaenoglyphis; specificEpithet: *villosa*; scientificNameAuthorship: (Hartig, 1841); **Location:** country: Germany; countryCode: DE; stateProvince: Baden-Württemberg; municipality: Karlsruhe; locality: Malsch, Luderbusch; verbatimElevation: 114 m; decimalLatitude: 48.9156; decimalLongitude: 8.3320; **Identification:** identifiedBy: Mar Ferrer-Suay; dateIdentified: 2023; **Event:** eventID: 1397; samplingProtocol: Malaise trap; eventDate: 2020-4/5-26/3; year: 1841; habitat: pond, gravel bank, salix bush; **Record Level:** language: en; institutionID: ZFMK; collectionID: ZFMK-TIS-2641209; basisOfRecord: PreservedSpecimen
- **Type status:** Other material. **Occurrence:** recordedBy: Doczkal, D.; individualCount: 1; sex: female; disposition: in collection; occurrenceID: 7CFBA8D2-C7AD-5902-8BCB-85C216AE519E; **Taxon:** family: Figitidae; genus: Phaenoglyphis; specificEpithet: *villosa*; scientificNameAuthorship: (Hartig, 1841); **Location:** country: Germany; countryCode: DE; stateProvince: Baden-Württemberg; municipality: Karlsruhe; locality: Malsch, Hansjakobstraße; verbatimElevation: 120 m; decimalLatitude: 48.8835; decimalLongitude: 8.3197; **Identification:** identifiedBy: Mar Ferrer-Suay; dateIdentified: 2023; **Event:** eventID: 804; samplingProtocol: Malaise trap; eventDate: 2020-4/5-26/10; year: 1841; habitat: garden; **Record Level:** language: en; institutionID: ZFMK; collectionID: ZFMK-TIS-2641217; basisOfRecord: PreservedSpecimen
- **Type status:** Other material. **Occurrence:** recordedBy: Doczkal, D.; individualCount: 1; sex: female; disposition: in collection; occurrenceID: E0A0AB79-2843-5B6F-BAF0-02E2FAD4CEE3; **Taxon:** family: Figitidae; genus: Phaenoglyphis; specificEpithet: *villosa*; scientificNameAuthorship: (Hartig, 1841); **Location:** country: Germany; countryCode: DE; stateProvince: Baden-Württemberg; municipality: Karlsruhe; locality: Malsch, Hansjakobstraße; verbatimElevation: 120 m; decimalLatitude: 48.8835; decimalLongitude: 8.3197; **Identification:** identifiedBy: Mar Ferrer-Suay; dateIdentified: 2023; **Event:** eventID: 804; samplingProtocol: Malaise trap; eventDate: 2020-4/5-26/10; year: 1841; habitat: garden; **Record Level:** language: en; institutionID: ZFMK; collectionID: ZFMK-TIS-2641218; basisOfRecord: PreservedSpecimen
- **Type status:** Other material. **Occurrence:** recordedBy: Doczkal, D.; individualCount: 1; sex: female; disposition: in collection; occurrenceID: 5B3BEAA4-EEB9-50BE-AA1B-A9DB3298784F; **Taxon:** family: Figitidae; genus: Phaenoglyphis; specificEpithet: *villosa*; scientificNameAuthorship: (Hartig, 1841); **Location:** country: Germany; countryCode: DE; stateProvince: Baden-Württemberg; municipality: Karlsruhe; locality: Malsch, Hansjakobstraße; verbatimElevation: 120 m; decimalLatitude: 48.8835; decimalLongitude: 8.3197; **Identification:** identifiedBy: Mar Ferrer-Suay; dateIdentified: 2023; **Event:** eventID: 804; samplingProtocol: Malaise trap; eventDate: 2020-4/5-26/10; year: 1841; habitat: garden; **Record Level:** language: en; institutionID: ZFMK; collectionID: ZFMK-TIS-2641220; basisOfRecord: PreservedSpecimen
- **Type status:** Other material. **Occurrence:** recordedBy: DINA; individualCount: 1; sex: male; disposition: in collection; occurrenceID: 150B367C-64E1-5377-A0DE-082E66D0AC7B; **Taxon:** family: Figitidae; genus: Phaenoglyphis; specificEpithet: *villosa*; scientificNameAuthorship: (Hartig, 1841); **Location:** country: Germany; countryCode: DE; stateProvince: Mecklenburg-Vorpommern; municipality: Greifswald; locality: Nat. res. Insel Koos; verbatimElevation: -1 m; decimalLatitude: 54.1694; decimalLongitude: 13.3690; **Identification:** identifiedBy: Mar Ferrer-Suay; dateIdentified: 2023; **Event:** eventID: 933; samplingProtocol: Malaise trap; eventDate: 2020-5-30; year: 1841; **Record Level:** language: en; institutionID: EVK; collectionID: ZFMK-TIS-2641221; basisOfRecord: PreservedSpecimen
- **Type status:** Other material. **Occurrence:** recordedBy: DINA; individualCount: 1; sex: female; disposition: in collection; occurrenceID: 31794BBB-2E5B-5AB1-B6FE-574F37A281B3; **Taxon:** family: Figitidae; genus: Phaenoglyphis; specificEpithet: *villosa*; scientificNameAuthorship: (Hartig, 1841); **Location:** country: Germany; countryCode: DE; stateProvince: Mecklenburg-Vorpommern; municipality: Greifswald; locality: Nat. res. Insel Koos; verbatimElevation: -1 m; decimalLatitude: 54.1694; decimalLongitude: 13.3690; **Identification:** identifiedBy: Mar Ferrer-Suay; dateIdentified: 2023; **Event:** eventID: 933; samplingProtocol: Malaise trap; eventDate: 2020-5-30; year: 1841; **Record Level:** language: en; institutionID: EVK; collectionID: ZFMK-TIS-2641222; basisOfRecord: PreservedSpecimen
- **Type status:** Other material. **Occurrence:** recordedBy: DINA; individualCount: 1; sex: female; disposition: in collection; occurrenceID: 75A4827D-F6C0-5879-A86D-9C0BA0016B6A; **Taxon:** family: Figitidae; genus: Phaenoglyphis; specificEpithet: *villosa*; scientificNameAuthorship: (Hartig, 1841); **Location:** country: Germany; countryCode: DE; stateProvince: Mecklenburg-Vorpommern; municipality: Greifswald; locality: Nat. res. Insel Koos; verbatimElevation: -1 m; decimalLatitude: 54.1694; decimalLongitude: 13.3690; **Identification:** identifiedBy: Mar Ferrer-Suay; dateIdentified: 2023; **Event:** eventID: 933; samplingProtocol: Malaise trap; eventDate: 2020-5-30; year: 1841; **Record Level:** language: en; institutionID: EVK; collectionID: ZFMK-TIS-2641223; basisOfRecord: PreservedSpecimen
- **Type status:** Other material. **Occurrence:** recordedBy: Vogel, Jonathan; individualCount: 1; sex: male; disposition: in collection; occurrenceID: 7211E815-0770-514E-8E97-3AC6C6FCABD3; **Taxon:** family: Figitidae; genus: Phaenoglyphis; specificEpithet: *villosa*; scientificNameAuthorship: (Hartig, 1841); **Location:** country: Germany; countryCode: DE; stateProvince: North Rhine-Westphalia; municipality: Bonn; locality: ZFMK garden; verbatimElevation: 68 m; decimalLatitude: 50.7218; decimalLongitude: 7.1132; **Identification:** identifiedBy: Mar Ferrer-Suay; dateIdentified: 2023; **Event:** eventID: 1782; samplingProtocol: sweep net; eventDate: 2020-9-15; year: 1841; **Record Level:** language: en; institutionID: ZFMK; collectionID: ZFMK-TIS-2641237; basisOfRecord: PreservedSpecimen
- **Type status:** Other material. **Occurrence:** recordedBy: Vogel, Jonathan; individualCount: 1; sex: female; disposition: in collection; occurrenceID: 4D747275-939D-5149-ADFB-91F88B00E75C; **Taxon:** family: Figitidae; genus: Phaenoglyphis; specificEpithet: *villosa*; scientificNameAuthorship: (Hartig, 1841); **Location:** country: Germany; countryCode: DE; stateProvince: North Rhine-Westphalia; municipality: Rhein-Sieg-Kreis; locality: Alfter, Möthengasse; verbatimElevation: 87 m; decimalLatitude: 50.7365; decimalLongitude: 7.0120; **Identification:** identifiedBy: Mar Ferrer-Suay; dateIdentified: 2023; **Event:** eventID: 778; samplingProtocol: sweep net; eventDate: 2021-5-21; year: 1841; habitat: private lawn; **Record Level:** language: en; institutionID: ZFMK; collectionID: ZFMK-TIS-2641263; basisOfRecord: PreservedSpecimen
- **Type status:** Other material. **Occurrence:** recordedBy: DINA; individualCount: 1; sex: female; disposition: in collection; occurrenceID: 2F7C913F-FFA7-5D2D-AC55-4544D0923048; **Taxon:** family: Figitidae; genus: Phaenoglyphis; specificEpithet: *villosa*; scientificNameAuthorship: (Hartig, 1841); **Location:** country: Germany; countryCode: DE; stateProvince: Thuringia; municipality: Schmalkalden, Meiningen; locality: Nat. res. Hofberg; verbatimElevation: 408 m; decimalLatitude: 50.6959; decimalLongitude: 10.2313; **Identification:** identifiedBy: Mar Ferrer-Suay; dateIdentified: 2023; **Event:** eventID: 925; samplingProtocol: Malaise trap; eventDate: 2020-5-30; year: 1841; **Record Level:** language: en; institutionID: EVK; collectionID: ZFMK-TIS-2641271; basisOfRecord: PreservedSpecimen
- **Type status:** Other material. **Occurrence:** recordedBy: DINA; individualCount: 1; sex: female; disposition: in collection; occurrenceID: DDEEF32F-F3DA-5BF5-A830-059D1BC825BB; **Taxon:** family: Figitidae; genus: Phaenoglyphis; specificEpithet: *villosa*; scientificNameAuthorship: (Hartig, 1841); **Location:** country: Germany; countryCode: DE; stateProvince: Mecklenburg-Vorpommern; municipality: Greifswald; locality: Nat. res. Insel Koos; verbatimElevation: -1 m; decimalLatitude: 54.169; decimalLongitude: 13.3691; **Identification:** identifiedBy: Mar Ferrer-Suay; dateIdentified: 2023; **Event:** eventID: 931; samplingProtocol: Malaise trap; eventDate: 2020-5-30; year: 1841; **Record Level:** language: en; institutionID: EVK; collectionID: ZFMK-TIS-2641273; basisOfRecord: PreservedSpecimen
- **Type status:** Other material. **Occurrence:** recordedBy: DINA; individualCount: 1; sex: female; disposition: in collection; occurrenceID: 0AA25F4B-567A-50A0-87B3-EB394EB0FE34; **Taxon:** family: Figitidae; genus: Phaenoglyphis; specificEpithet: *villosa*; scientificNameAuthorship: (Hartig, 1841); **Location:** country: Germany; countryCode: DE; stateProvince: Mecklenburg-Vorpommern; municipality: Greifswald; locality: Nat. res. Insel Koos; verbatimElevation: -1 m; decimalLatitude: 54.169; decimalLongitude: 13.3691; **Identification:** identifiedBy: Mar Ferrer-Suay; dateIdentified: 2023; **Event:** eventID: 931; samplingProtocol: Malaise trap; eventDate: 2020-5-30; year: 1841; **Record Level:** language: en; institutionID: EVK; collectionID: ZFMK-TIS-2641274; basisOfRecord: PreservedSpecimen
- **Type status:** Other material. **Occurrence:** recordedBy: DINA; individualCount: 1; sex: female; disposition: in collection; occurrenceID: 3FB8E391-DBEC-55A1-B84A-80941245288B; **Taxon:** family: Figitidae; genus: Phaenoglyphis; specificEpithet: *villosa*; scientificNameAuthorship: (Hartig, 1841); **Location:** country: Germany; countryCode: DE; stateProvince: Thuringia; municipality: Erfurt; locality: Nat. res. Schwellenburg; verbatimElevation: 203 m; decimalLatitude: 51.03; decimalLongitude: 10.9549; **Identification:** identifiedBy: Mar Ferrer-Suay; dateIdentified: 2023; **Event:** eventID: 940; samplingProtocol: Malaise trap; eventDate: 2020-5-30; year: 1841; **Record Level:** language: en; institutionID: EVK; collectionID: ZFMK-TIS-2641279; basisOfRecord: PreservedSpecimen
- **Type status:** Other material. **Occurrence:** recordedBy: Taxon Expeditions Team; individualCount: 1; sex: female; disposition: in collection; occurrenceID: 9B3553F6-6B91-574E-AC16-5E91706C9B05; **Taxon:** family: Figitidae; genus: Phaenoglyphis; specificEpithet: *villosa*; scientificNameAuthorship: (Hartig, 1841); **Location:** country: The Netherlands; countryCode: NL; stateProvince: Noord-Holland; municipality: Amsterdam; locality: Vondelpark; verbatimElevation: 2 m; decimalLatitude: 52.3581; decimalLongitude: 4.8681; **Identification:** identifiedBy: Mar Ferrer-Suay; dateIdentified: 2023; **Event:** eventID: 1428; samplingProtocol: Malaise trap; eventDate: 2019-6-21/25; year: 1841; **Record Level:** language: en; institutionID: ZFMK; collectionID: ZFMK-TIS-2641296; basisOfRecord: PreservedSpecimen
- **Type status:** Other material. **Occurrence:** recordedBy: Doczkal, Dieter;Voith, J.; individualCount: 1; sex: female; disposition: in collection; occurrenceID: 955A1D42-85A3-5818-8E30-52304C877207; **Taxon:** family: Figitidae; genus: Phaenoglyphis; specificEpithet: *villosa*; scientificNameAuthorship: (Hartig, 1841); **Location:** country: Germany; countryCode: DE; stateProvince: Bavaria; municipality: Garmisch-Partenkirchen; locality: Zugspitze, Platt; verbatimElevation: 1980 m; decimalLatitude: 47.4053; decimalLongitude: 11.0091; **Identification:** identifiedBy: Mar Ferrer-Suay; dateIdentified: 2023; **Event:** eventID: 1425; samplingProtocol: Malaise trap; eventDate: 2018-7-5/18; year: 1841; habitat: mountain; **Record Level:** language: en; institutionID: ZFMK; collectionID: ZFMK-TIS-2641297; basisOfRecord: PreservedSpecimen

#### Diagnosis

Antennae of both sexes with rhinaria beginning on F3, pedicel as long as F1, F1 subequal to F2, F2 shorter than F3, F3 shorter than F4 (Fig. 3f); pronotal carinae present, notauli absent, scutellum with two deep foveae, oval, more or less separated by median carina or completely fused (Fig. 4f), propodeal carinae present; radial cell partially open, 2.1-2.7 times as long as wide.

##### Molecular characterisation

Maximum barcode-distance within species: 2.6% (37).

Minimum barcode-distance to closest species: 6.3% (*P.belizini*).

Consensus barcode sequence (652 bp):

5’- AATTTTATATTTTATTTTTGGAATTTGGTCAGGAATAATTGGCTCTGCATTAAGAATAATTATTCGTATAGAATTAGGGACTCCTTCACAATTTATTGGGAATGATCAAATTTATAATTCAATTGTGACAGCTCATGCTTTTATTATAATTTTTTTTATAGTGATACCTATTATAGTTGGAGGATTTGGTAATTATTTAGTCCCTTTAATATTATCAGCACCAGATATAGCGTTCCCTCGTCTTAATAATATAAGATACTGATTATTATTACCAGCATTAATTTTATTAGTTTCAAGAATATTTATTGATCAAGGGGCAGGAACAGGATGAACAGTTTATCCACCTTTATCTTCTAATTTAAGACATTCAGGAATTTCAGTTGATTTAACAATTTTTGCTTTACATTTAAGGGGGGTTTCTTCAATTTTAGGGTCAATTAATTTTATTACTACAATTTTAAATATACGAATTATTTCAATAGATAAAATTTCTTTATTTATTTGGTCTATTTTCCTAACAACAATTTTATTATTATTATCTTTACCGGTTCTAGCTGGAGGAATTACAATATTATTATTTGATCGTAATATAAATACTTCTTTTTTTGACCCTATAGGAGGAGGGGATCCAATTTTATACCAACATTTATTT -3’

#### Distribution

Cosmopolitan (Ferrer-Suay et al. 2023).

#### Taxon discussion

*P.villosa* is reported to be morphologically considerably variable (Pujade-Villar et al. 2007). Here, the second-ranked partition of the ASAP species delimitation algorithm infers four separate entities within *P.villosa*. All other algorithms infer the clusters as conspecific. As we can neither find consistent morphological traits to separate the putative species nor find any geographic or temporal (collecting months) patterns within and between the molecular clusters, we treat all included specimens as belonging to *P.villosa*. The high intraspecific variability in both morphology and molecular data might, however, indicate a cryptic species complex behind *P.villosa* that requires more in-depth studies.

### Phaenoglyphis xanthochroa

Förster, 1869

ECCE8592-7D62-5483-9910-1DC0AA421773

#### Materials

- **Type status:** Other material. **Occurrence:** recordedBy: GBOL III; individualCount: 1; sex: female; disposition: in collection; occurrenceID: F7C172B0-1FB9-5483-8FBB-7BFC0D8FCAD5; **Taxon:** family: Figitidae; genus: Phaenoglyphis; specificEpithet: *xanthochroa*; scientificNameAuthorship: Förster, 1869; **Location:** country: Germany; countryCode: DE; stateProvince: Hesse; municipality: Waldeck-Frankenberg; locality: National park Kellerwald-Edersee, Banfehaus; verbatimElevation: 265 m; decimalLatitude: 51.167; decimalLongitude: 8.9749; **Identification:** identifiedBy: Mar Ferrer-Suay; dateIdentified: 2023; **Event:** eventID: 1078; samplingProtocol: Malaise trap (Krefeld type); eventDate: 2021-7/8-22/5; year: 1869; habitat: old floodplain of the Banfe; **Record Level:** language: en; institutionID: ZFMK; collectionID: ZFMK-TIS-2632857; basisOfRecord: PreservedSpecimen
- **Type status:** Other material. **Occurrence:** recordedBy: GBOL III; individualCount: 1; sex: female; disposition: in collection; occurrenceID: BEA0E06B-AE5C-5D91-83C0-77F79133F67B; **Taxon:** family: Figitidae; genus: Phaenoglyphis; specificEpithet: *xanthochroa*; scientificNameAuthorship: Förster, 1869; **Location:** country: Germany; countryCode: DE; stateProvince: Hesse; municipality: Waldeck-Frankenberg; locality: National park Kellerwald-Edersee, Maierwiesen; verbatimElevation: 365 m; decimalLatitude: 51.1555; decimalLongitude: 9.0015; **Identification:** identifiedBy: Mar Ferrer-Suay; dateIdentified: 2023; **Event:** eventID: 1033; samplingProtocol: Malaise trap (Krefeld type); eventDate: 2021-6/7-22/8; year: 1869; **Record Level:** language: en; institutionID: ZFMK; collectionID: ZFMK-TIS-2641256; basisOfRecord: PreservedSpecimen

#### Diagnosis

*Phaenoglyphisxanthochroa* is easily differentiated from the other *Phaenoglyphis* species by its dark yellow body and deeply excavated notauli (Fig. 4g).

##### Molecular characterisation

Maximum barcode-distance within species: 2.3% (2).

Minimum barcode-distance to closest species: 10.7% (*P.villosa*).

Consensus barcode sequence (652 bp):

5’- GATTTTATATTTTATTTTTGGGATTTGGTCAGGAATAATTGGCTCAGCTTTAAGAATAATTATTCGAATAGAATTAGGAACCCCTTCTCAATTGATTGGTAATGATCAAATTTATAATTCAATTGTAACAGCTCATGCTTTTATTATAATTTTTTTTATAGTTATACCAATTATAGTAGGTGGGTTTGGGAATTATTTAATTCCTTTAATATTATCAGCCCCTGATATAGCTTTCCCACGTTTAAATAATATAAGATTTTGGTTATTAATCCCAGCTTTATTTCTATTAATTATAAGAATATTTATTGATCAAGGGGCAGGGACTGGATGAACTGTTTACCCTCCTTTATCTTCAAATTTAGGTCATTCTGGGATTTCTGTTGATTTAACAATTTTTTCACTTCATTTAAGAGGAGTATCTTCAATTTTAGGGGCAATTAATTTTATTTCAACAATTTTAAATATACGAATTATTARAATAGATAAAATTTCATTATTTATTTGATCAATTTTTTTAACAACAATTTTATTATTATTGTCTTTACCTGTTTTAGCTGGAGGTATTACTATATTATTATTTGATCGAAATTTAAATACTTCTTTTTTTGACCCTATAGGAGGAGGAGACCCAATTTTATACCAACATTTATTT-3’

#### Distribution

Austria, Czech Republic, Finland, France, Germany, Ireland, Poland, Sweden, Switzerland, The Netherlands, and United Kingdom: England (Ferrer-Suay et al. 2023).

#### Taxon discussion

The sequence with the BOLD-ID AMTPB279-15 has an associated photograph uploaded on BOLD. The specimen shown exhibits the unique morphology of *P.xanthochroa* and the identity is further confirmed by an expert hymenopterist. These circumstances led us to include the specimen into the molecular characterisation of the species. *Phaenoglyphisxanthochroa* is so unique in morphology and shows a large distance to the barcode sequences of other *Phaenoglyphis* species that it leaves room for debate whether to put this species in its own genus. We refrain from doing so as we think that a more thorough molecular dataset needs to back up this decision and the practical use of a monotypic genus is very limited.

## Analysis

Out of 55 specimens processed, 48 barcode sequences were generated (87% success rate). Including the publicly available barcodes from BOLD, the final dataset consists of 101 ingroup and three outgroup sequences.

**Figure 2.**
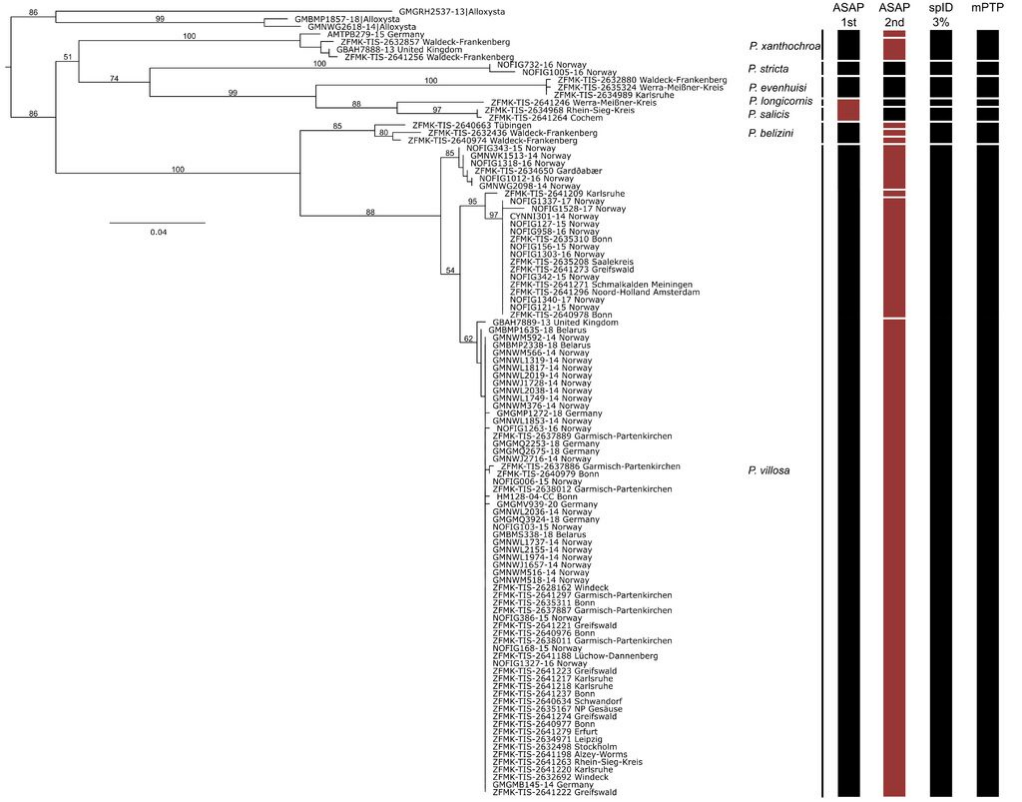
Maximum Likelihood tree, based on CO1 barcode data (produced with IQ Tree) with ultrafast bootstrap values shown on the branches. The clusters of the species delimitation algorithms are shown to the right, summarised as bars (ASAP 1^st^ and 2^nd^, multirate PTP and Species Identifier with a threshold of 3%). The black bars indicate delimitation results that match our morphological identifications, while red bars represent conflicts between morphology and molecular species delimitation.

**Figure 3.**
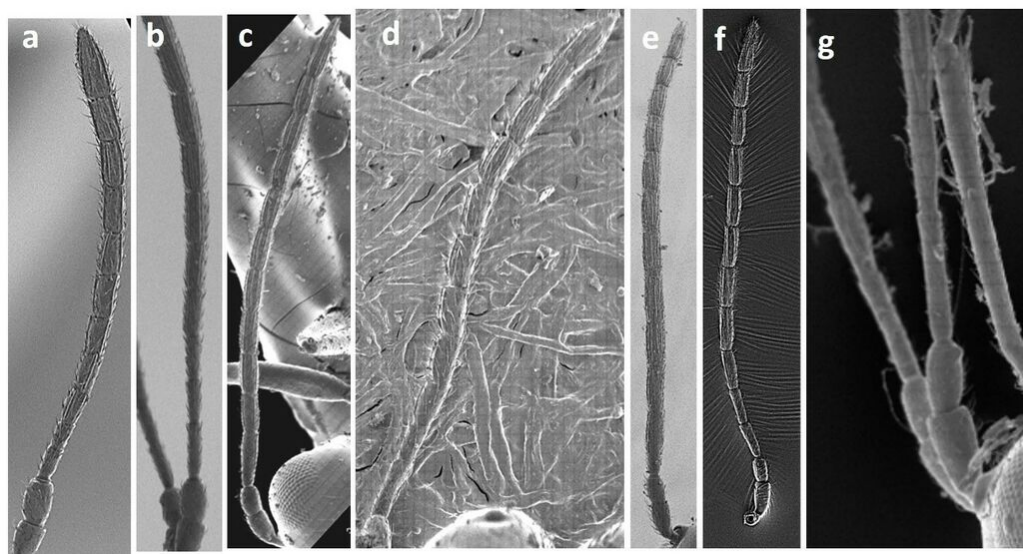
SEM images of female *Phaenoglyphis* antennae (all images show corresponding holotypes): **a** *P.belizini*; **b** *P.evenhuisi*; **c** *P.longicornis*; **d** *P.salicis*; **e** *P.stricta*; **f** *P.villosa*; **g** *P.xanthochroa*. Images are taken from Ferrer-Suay et al. (2019).

**Figure 4.**
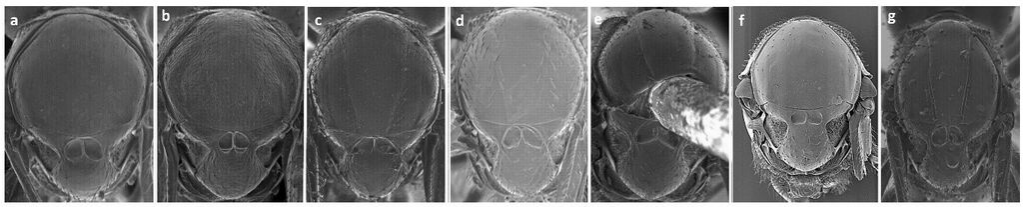
SEM images of *Phaenoglyphis* mesoscutum (dorsal view; all images show corresponding holotypes): **a** *P.belizini*; **b** *P.evenhuisi*; **c** *P.longicornis*; **d** *P.salicis*; **e** *P.stricta*; **f** *P.villosa*; **g** *P.xanthochroa*. Images are taken from Ferrer-Suay et al. (2019).

The species limits established by morphological features are corroborated by the molecular results (Fig. 2). Seven *Phaenoglyphis* species have been identified, which is supported by the molecular species delimitations of Species Identifier, mPTP and largely conclusive with the first-ranked partition of ASAP (except *Phaenoglyphislongicornis* and *P.salicis* being clustered as conspecific). The second-ranked partition of the ASAP analysis split the included specimens into 13 separate species. The first and second-ranked partition of ASAP had an identical ASAP-score (4.0) and were, therefore, included both in Fig. 2.

## Discussion

We complement the previously-established morphological characterisation of the genus *Phaenoglyphis* and seven of its species from north-western Europe (Ferrer-Suay et al. 2018) with the first molecular characterisation of their respective DNA barcode sequences: *P.belizini*, *P.evenhuisi*, *P.longicornis*, *P.salicis*, *P.stricta*, *P.villosa*, and *P.xanthochroa*. This currently leaves ten species known from north-western Europe without molecular characterisation (*P.abbreviata*, *P.americana*, *P.calverti*, *P.fuscicornis*, *P.gutierrezi*, *P.heterocera*, *P.nigripes*, *P.proximus*, *P.pubicollis* and *P.ruficornis*).

We complement BOLD with additional sequences for all of the molecularly characterised taxa, except *P.stricta*, for which we did not provide any additional sequences. Four of the taxa within our material were not represented on BOLD before (*P.belizini*, *P.evenhuisi*, *P.longicornis*, and *P.salicis*).

Our discovery of *P.belizini* and *P.evenhuisi* in our material represents new records for Germany.

The results of the mPTP and SpID species delimitations were largely congruent with our morphological identifications. There is an apparent over-splitting by the second-ranked ASAP partition and one case of lumping in the first-ranked ASAP partition. This could be interpreted as additional evidence that it is advisable to use more than one species delimitation algorithm and is in line with previous findings (e.g. Meier et al. (2022)).

Ten species from north-western Europe are currently lacking CO1 barcode sequences and these can hopefully be added in future investigations. It is important to note that, within Charipinae, *Alloxysta* specimens are the most common and they are very well represented in many samples, but *Phaenoglyphis* is comparably rare, which makes it more difficult to acquire a good number of fresh specimens for sequencing.

The 6 bp deletion, present exclusively in the *Phaenoglyphis* barcodes within Charipinae, is additional evidence for the monophyly of the genus that was previously questioned (Paretas-Martínez et al. 2007). More extensive analyses, ideally based on a phylogenomics/taxonomics dataset, are needed to answer this question and steer the classification of monophyletic Charipinae genera.

Molecular characterisation of Charipinae species is still at its first steps. With this study, a significant portion of one of the main genera, *Phaenoglyphis*, is now ready to be included in DNA barcode-based activities. However, many species remain uncharacterised and it will be necessary to continue integrative taxonomy studies and to improve our knowledge of the genus.

## Supplementary Material

XML Treatment for Phaenoglyphis

XML Treatment for Phaenoglyphis belizini

XML Treatment for Phaenoglyphis evenhuisi

XML Treatment for Phaenoglyphis longicornis

XML Treatment for Phaenoglyphis salicis

XML Treatment for Phaenoglyphis stricta

XML Treatment for Phaenoglyphis villosa

XML Treatment for Phaenoglyphis xanthochroa

B82FB9B5-5994-54EC-ACCC-18DE59E68DF8 10.3897/BDJ.12.e120950.suppl1 Supplementary material 1 BOLD sequence IDs of all specimens used for the molecular analysis Data type BOLD sequence IDs Brief description The table lists all specimens used for the molecular species delimitation methods, including the specimens studied morphologically, that were used to molecularly characterise the genus and each species. File: oo_1014243.xlsx https://binary.pensoft.net/file/1014243 Vogel, Jonathan

4A37C752-BD4E-5B12-A409-FBF17C6DFD76 10.3897/BDJ.12.e120950.suppl2 Supplementary material 2 CO1 barcode distance matrix Data type genetic distances Brief description The data matrix showing the CO1 barcode sequence distances between all individuals that were studied morphologically. This table is the basis for the molecular characterisation of the species. File: oo_1014291.csv https://binary.pensoft.net/file/1014291 Vogel, Jonathan
